# Biopolymers Hybrid Particles Used in Dentistry

**DOI:** 10.3390/gels7010031

**Published:** 2021-03-22

**Authors:** I-Hao Chen, Tzer-Min Lee, Chih-Ling Huang

**Affiliations:** 1 School of Dentistry, Kaohsiung Medical University, Kaohsiung 807, Taiwan; a0929000761@gmail.com; 2 Institute of Oral Medicine, College of Medicine, National Cheng Kung University, Tainan 701, Taiwan; 3 School of Dentistry, College of Medicine, National Cheng Kung University, Tainan 701, Taiwan; 4 Center for Fundamental Science, Kaohsiung Medical University, Kaohsiung 807, Taiwan

**Keywords:** biopolymer gels, nano-particles, biomedical applications, dentistry

## Abstract

This literature review provides an overview of the fabrication and application of biopolymer hybrid particles in dentistry. A total of 95 articles have been included in this review. In the review paper, the common inorganic particles and biopolymers used in dentistry are discussed in general, and detailed examples of inorganic particles (i.e., hydroxyapatite, calcium phosphate, and bioactive glass) and biopolymers such as collagen, gelatin, and chitosan have been drawn from the scientific literature and practical work. Among the included studies, calcium phosphate including hydroxyapatite is the most widely applied for inorganic particles used in dentistry, but bioactive glass is more applicable and multifunctional than hydroxyapatite and is currently used in clinical practice. Today, biopolymer hybrid particles are receiving more attention as novel materials for several applications in dentistry, such as drug delivery systems, bone repair, and periodontal regeneration surgery. The literature published on the biopolymer gel-assisted synthesis of inorganic particles for dentistry is somewhat limited, and therefore, this article focuses on reviewing and discussing the biopolymer hybrid particles used in dentistry.

## 1. Introduction

Biopolymer gels are widely used in biomedical applications [1,2,3]. Examples include tissue engineering [4,5], wound dressing [6,7], dentistry [8] and in particular, the combination of biopolymers with nanoparticles to fabricate membranes for periodontal regeneration [9,10]. According to the different raw materials and production methods, there are many different commercial products of biopolymers, such as polylactic acid, collagen, gelatin, and so on [11]. Periodontal regeneration membranes made of collagen are widely used in dentistry, i.e., the polylactide and polyglycolide membrane (Atrisorb^®^, and Resolut LT^®^) [12], bovine type I collagen membrane (Osseoguard™), porcine type I and III collagen membrane (BioGide^®^) [13], resorbable collagen membrane (Biomend^®^), and the gelatin-based sponge (Gelfoam^®^) [14].

The association between biopolymer and hybrid particles is important for many biomedical functions, such as antimicrobial applications [15] (including drug delivery [16]) and guided bone regeneration [17,18]. This system has been emphasized by many researchers and clinicians, and it provides a useful framework to improve the repair of patients. The common limitations of biopolymer are that the mechanical strength is insufficient, so various crosslinking techniques have been applied to promote the mechanical properties of biopolymers for different biotechnological applications [19]. In the case of biodegradable materials, the small molecules that are degraded sometimes also produce cytotoxicity. For example, acidic small molecules can easily cause inflammation when polylactic acid is degraded [20]. If the solvents (acids or other organic solvents) used in the production process cannot be completely removed, this makes the biocompatibility of biopolymers worse than expected. Of course, the production cost is also a factor that must be considered, and novel methods with high efficiency as compared to conventional solvent extraction were developed [1].

In this article, we focus on biopolymer hybrid particles in dentistry and their potential role in guided tissue regeneration membranes. The article is organized as follows. In Section 2, we describe the role of inorganic particles in dentistry, especially in recent research on guided tissue regeneration membrane or drug delivery, which are common topics in clinical applications. In Section 3, we describe biopolymer hybrid particles regarding their promising advantages for use in dentistry. The conclusions and outlook are drawn in Section 4.

## 2. Inorganic Particles Used in Dentistry

Inorganic particles play an important role in dentistry, especially dental resins. They are composite materials consisting of mostly inorganic fillers and additives bound together with a polymer matrix [21]. Some inorganic particles are used for preventive oral disease drugs and implants, so their chemical, physical, and biological properties must be considered. It is necessary to simultaneously satisfy the mechanical properties and bioactivity to repair bone defects in dentistry. Inorganic particles are commonly used in ceramics, such as hydroxyapatite [22], calcium phosphate [23], and bioactive glass [24].

### 2.1. Hydroxyapatite

Hydroxyapatite is a primary constituent of bones and teeth in vertebrates and is known for its biocompatible, bioactive, osteoconductive, non-toxic, non-inflammatory, and non-immunogenic properties [25]. Hydroxyapatite can be synthesized in various ways, including via dry, wet, and high-temperature methods [26]. By choosing different methods and manipulating operating parameters properly, we can synthesize tailor-made hydroxyapatite nanoparticles. The precursor of synthesized hydroxyapatite can be derived from chemical or natural resources [27]. Although the pure form of the precursors can usually increase purity, hydroxyapatites derived from natural resources are more economic and show better metabolic activity and enhanced bioactivity in comparison to their synthetic counterparts [3]. Suitable natural resources include eggshells [28], bovine bones [29], fish scales [30], etc.

Combining hydroxyapatite with organic or inorganic compounds can enhance the mechanical strength, biocompatibility, and bioactivity, as well as some specific features [31,32]. Polyurethane membranes fabricated with titanium dioxide (TiO_2_) and hydroxyapatite has higher water contact angle, mechanical strength, and stiffness than those without TiO_2_ and hydroxyapatite. They also show better cell adhesion, viability, proliferation, alkaline phosphatase (ALP) activity, and calcium content [33]. Guided tissue membranes embedded with hydroxyapatite have good biocompatibility and osteoconductivity and can establish desirable conditions to prompt injured bone regeneration [34]. Hydroxyapatite also has potential in dental tissue regeneration.

The minerals in the natural hard tissue are mainly composed of hydroxyapatite and type I collagen. The inorganic compounds like calcium and hydroxyapatite precipitate in the organic matrix. Their nucleation, crystal growth, morphology, and orientation is regulated by interaction with the organic matrix, which is called biomineralization. These nanohybrid structures provide excellent strength and resilience against outside pressure. Biopolymer gel-assisted synthesis for hydroxyapatite particles is developed via biomimetic mineralization [35] using biopolymers such as collagen [36], gelatin [37], chitosan [38], etc.

#### 2.1.1. Collagen Gel-Assisted Synthesis

Collagen has the highest amount of protein in the animal kingdom and is a key component of the extracellular matrix. Collagen in the human body is categorized into 3 types: type I, II, or III, and type I is commonly used in tissue engineering. Collagen type I is made up of three alpha chains, and each chain consists of glycine, proline and hydroxyproline, respectively [39].

Collagen has good biological activity, and cell affinity and has become a common wound dressing material in the market [40]. It is usually made from natural collagen extracted from beef tendon tissue and is cut into appropriate shapes for use according to different clinical needs. It has good biocompatibility, and has the effects of promoting hemostasis, wound healing, and tissue repair. Human bone cells seeded onto collagen and collagen/calcium phosphate coated substrates yielded well-developed filopodia and lamellipodia, and cell proliferation was significantly higher than in the other specimens [41]. It is suitable for general or surgical wound treatment, can support wound healing, and is also a commercial product used for dental periodontal regeneration film [42].

The limitation of collagen is its structural stability. In the process of extracting collagen, the assembly structure and natural cross-linking are often destroyed, weakening its mechanical strength, thermal stability, and enzyme resistance [8]. In addition, collagen does not have antibacterial and hemostatic effects, so it needs to be combined with other biopolymers, nano-particles, or drugs to improve its shortcomings [43]. The mechanical properties of collagen composite membranes can be reinforced by chitosan and beta-tricalcium phosphate [44]. With increasing chitosan and beta-tricalcium phosphate contents, the flexural-strength and tear-strength have been improved without a negative effect on cell morphology, viability, and proliferation.

**Figure 1:**
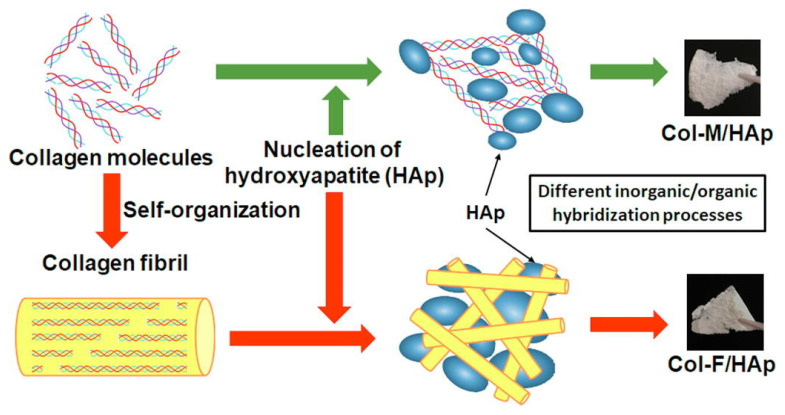
The fabrication processes for biomimetic collagen/hydroxyapatite hybrids [36]. Hydroxyapatite nanocrystals were both synthetized through the chemical reactions of the collagen molecular or fibril states with hydroxyapatite, indicating that the hydroxyapatite crystal growth was effectively enhanced by the collagen. Adapted with permission from Elsevier, Copyright (2019).

Chai et al. [36] compared two types of hybrid structures, hydroxyapatite precipitating to collagen molecules (Col-M/HAp) and hydroxyapatite precipitating to collagen fibrils (Col-F/HAp), to fish scales (shown in Figure 1). The aggregated and/or cross-linked states of Col-F/HAp have a higher density than Col-M/HAp and are close to those of the fish scale (i.e., natural hard tissue). In the Col-F/HAp, the collagen molecules will form the fibrils first, and the fibril surfaces will regulate the precipitated hydroxyapatite nanocrystals. Gelatin is produced by partial hydrolysis of collagen, which partially loses its triple-helix structures and has the same chemical composition as the organic bone matrix. However, its fast dissolution rate in physiological conditions and lack of pores with suitable dimensions for cell permeation limit the gelatin’s application.

#### 2.1.2. Gelatin Gel-Assisted Synthesis

Gelatin is mainly composed of protein and is an irreversibly hydrolyzed form of collagen. It is light-yellow and transparent gum, usually extracted from the skin, bones, and connective tissues of cattle, pigs, and fish. Gelatin can be used in gelling agents for food, medicine, or cosmetics [45]. Gelatin has various attractive features, such as biocompatibility, low immunogenicity, biodegradability, and ease of manipulation. It can be made as various microspheres to function as a drug delivery system [46].

The limitation of gelatin is its dissociation temperature around 30–35 °C and the fact that it dissolves at elevated temperatures while forming a swollen hydrogel below this phase change temperature. The physical properties of gelatin gel can be tuned physical, enzymatic, and cross-linking agents to modify its use as a drug delivery vehicle [47], wound dressing [48], or food packaging [49]. For example, the lower crosslinking results in rapid swelling and diffusion, but the higher crosslinking results in reduced swelling and sustained diffusion. This directly influences the efficiency of drug delivery.

**Figure 2:**
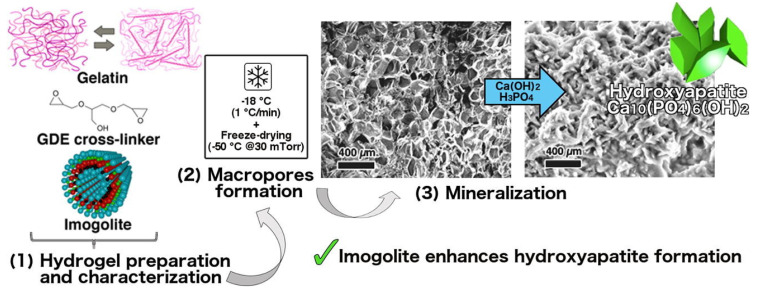
The formation of hydroxyapatites in gelatin/imogolite macroporous hydrogels [37]. Imogolite clay was used at the nucleation sites for the growth of calcium phosphates in gelatin-based hydrogels, and the presence of imogolites in the hydrogel promotes the formation of hydroxyapatites. (GDE: glycerol diglycidyl ether). Adapted with permission from Elsevier, Copyright (2018).

The chemical cross-linking agents used for gelatin gel-assisted synthesis include glutaraldehyde [50], genipin [51], and glycerol diglycidyl ether (GDE). Gelli et al. [37] added the GDE, a chemical cross-linking agent used for increasing stability at physiological temperature, to the gelatin solution (shown in Figure 2). Imogolite, a naturally occurring hydrous aluminosilicate that has nano-sized cavities, was then added to the gelatin solution to act as nucleation size for enhancing the formation of both hydroxyapatite and carbonated hydroxyapatite. After the gelatin solution was gelled, the gelatin hydrogel was obtained via a cryogenic formation procedure to acquire μm-sized porosity. The experimental findings of this gelatin hydrogel characterized by a multi-technique approach showed the desired pore size from 5 to 125 μm (100–125 μm required in materials for the regeneration of bone tissues) and promoted hydroxyapatite formation proved by means of field-emission scanning electron microscopy (FE-SEM), X-ray diffraction (XRD) analysis, and thermogravimetric analysis (TGA).

#### 2.1.3. Chitosan Gel-Assisted Synthesis

Chitosan is composed of glucosamine and derived via the deacetylation of chitin. Chitin is a natural polysaccharide with a structure like cellulose. It is composed of thousands of particles of N-acetylglucosamine, which is a kind of glucosamine ((1,4)-2-amino-2-deoxy-β-D-glucan) polysaccharide. Its sources are widely distributed and can be found in arthropods, mollusks, algae, and fungi, including the crustaceans of crabs, shrimps and other marine animals [52]. Chitosan is a product obtained by the deacetylation of chitin. It can be regarded as a polymer composed of N-acetylglucosamine and glucosamine. It is not a single specific structure. It can be deacetylated according to molecular weight. The degree of chemical conversion and the pH value of the solution vary. Depending on different processing methods, the degree of deacetylation of chitin may be between 65% and 99%, and generally between 70% and 80%. If the degree of acetyl removal is more than 65%, it can be recognized as chitosan. In addition, chitosan with a degree of deacetylation of more than 60% is soluble in weak acid [53].

Chitosan has a positively charged amine group (NH_3_^+^) while it is dissolved in weak acid solution (–NH_2_ + H^+^ → NH_3_^+^) [54]. The positively charged groups provide bacteriostasis and biocompatibility, as well as promoting cell growth. Compared with chitin, chitosan has a wider range of application [55]. It possesses good mechanical properties, biodegradability, biocompatibility, and antibacterial effects. Chitosan’s use as a wound dressing, the commercial product has been demonstrated by experiments (cytotoxicity and hemostatic activity) to show its high biocompatibility and non-toxicity [56]. In addition, chitosan has a positive charge (polyglucosamine; –NH_3_^+^), which can promote the rapid accumulation of negatively charged platelets and red blood cells, thereby causing blood to coagulate, which can quickly stop bleeding or control bleeding from wounds. Additionally, the positive electric energy on the surface produces an antibacterial effect for up to seven days, which can deter wound infections [57].

**Figure 3:**
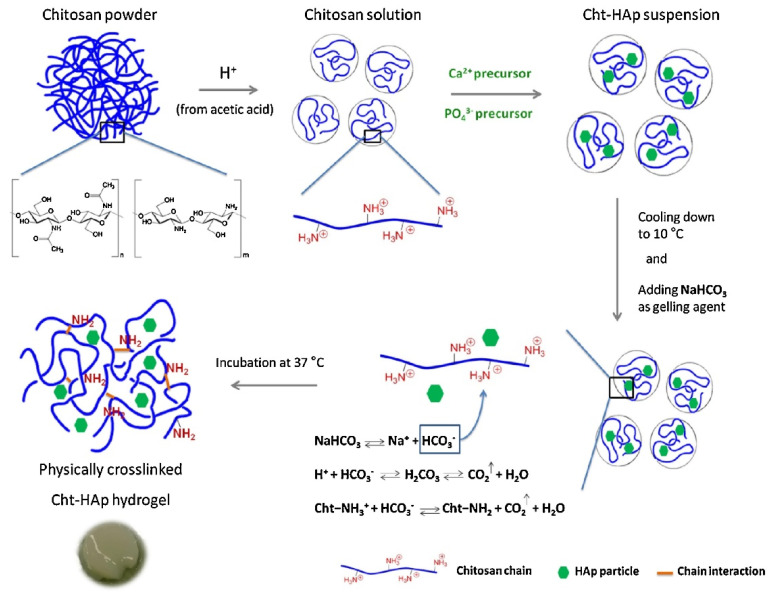
The preparation of physically crosslinked chitosan hydroxyapatite hydrogel [38]. Adapted with permission from Elsevier, Copyright (2017).

Rogina et al. [38] developed a pH-responsive-hydroxyapatite-based gel with sodium bicarbonate as the gelling agent (shown in Figure 3). This can gel within 4 min without excess sodium ion concentrations, and most important, it is non-cytotoxic. The in situ synthesis of the apatite phase facilitates the physical crosslinking by reducing the acidity of the chitosan solution, not only enhancing the mechanic properties of the gel but also solving the problem of a low pH value resulting from the synthesis of the chitosan-based hydrogel.

The incorporation of nano-hydroxyapatite with chitosan to fabricate composite material can be prepared via an in situ hybridization route [58]. Chitosan was dissolved as a polymer solution. A calcium nitrate tetrahydrate (Ca(NO_3_)_2_·4H_2_O) and diammonium hydrogen phosphate ((NH_4_)_2_HPO_4_) solution was added into the chitosan solution to obtain a homogeneous polymer solution. The white gelatinous precipitate was separated by the polymer solution. While increasing the chitosan, the aggregation of hydroxyapatite nanoparticles was enhanced. This indicated that chitosan had a strong adsorption interaction with hydroxyapatite, and the hydroxyapatite nanocrystals were aligned along the chitosan molecules. Observation via scanning electron microscopy noted that the hydroxyapatite nanoparticles had a uniform size distribution. The spherical inorganic hydroxyapatite was fabricated via chitosan gel-assisted synthesis.

### 2.2. Calcium Phosphate

Calcium phosphate can be successfully used as a filler for polymeric composites [59]. Calcium phosphate is one of the compositions of the inorganic phase of bone, and hence it possesses biocompatible properties, osteoconductivity, and osteoinductivity. The surface of calcium phosphate undergoes solution-mediated surface reactions when in contact with biological media, and this is partly responsible for new bone formation. There are several crystalline forms of calcium phosphate (alpha and beta forms) [60]. Hydroxyapatite and tricalcium phosphate (TCP) [61] are the forms of calcium phosphate which are mostly studied for bone tissue engineering [62]. Calcium phosphate nanotube arrays had a pronounced effect on cell attachment on the biological responses of human bone cells [63]. The calcium phosphate grooved patterns affect the human fetal osteoblast cell shape and cytoskeletal structure, thus influencing cell proliferation and cell adhesion forces [64].

The size and shape of calcium phosphate particles are critical for biological reactions such as osteoblast proliferation, cellular activity, apoptosis, and macrophage activity [65]. By choosing different synthetic methods, altering the starting concentration and Ca/P ratio of the precursor can achieve the expected size [66] and shape of calcium phosphate particles. The gelatin–strontium substituted calcium phosphate composites can be fabricated via coprecipitation in a gelatin solution to form unidirectional porous scaffolds with an oriented microtubular structure [67]. The incorporation of calcium phosphate into the guided tissue regeneration membrane can alter the membrane’s bioactivity, osteoconduction, and other properties like porosity [68], mechanical strength, pH value, and antibacterial ability. An inorganic crystal was guided via epitaxy with the organic matrix as a template.

**Figure 4:**
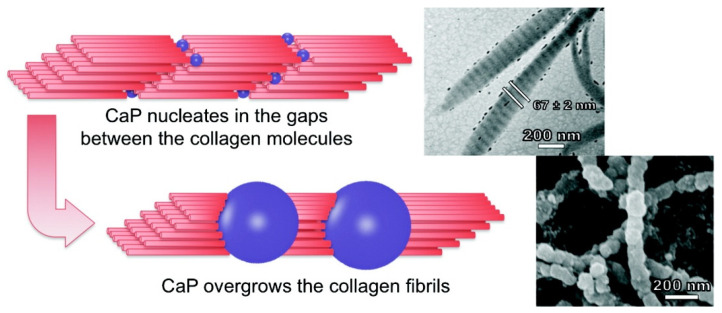
The preparation of mineralized nanofibers of collagen fibrils containing calcium phosphate [69]. This structure, which resembles the basic constituent of bones, assembles itself without the addition of non-collagenous proteins or their polymeric substitutes. Adapted with permission from American Chemical Society, Copyright (2017).

Maas et al. [69] prepared mineralized collagen fibrils closely resembling natural bone material by using a nano-porous polycarbonate track-etched membrane as a filtration membrane and substrate (shown in Figure 4). Feed solution containing 1 mg/mL collagen, 1–20 mM CaCl_2,_ and 1 mM HCL (pH value = 3.0) was pumped into the receiver solution containing 0.66 mM Na_2_PO_4_ and 1 mM NaOH under a gauge pressure of 250 mbar. With this method, the diameter of the mineralized fibril can be controlled through the choice of the size of the nanopores in the membrane separating the feed solution from the receiver solution.

**Figure 5:**
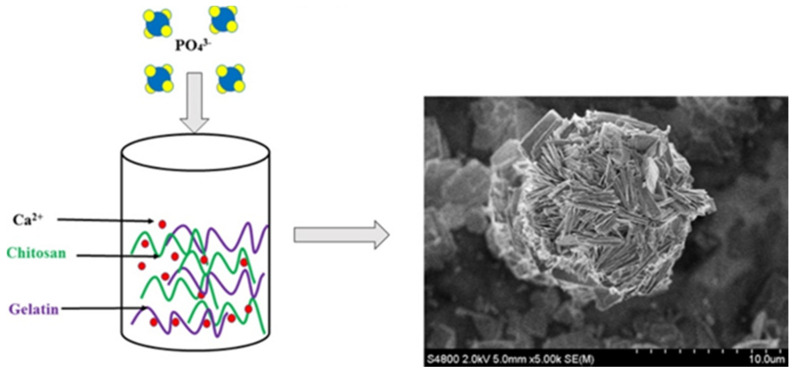
Gelatin–chitosan guided crystal growth to form nano-porous supramolecular calcium phosphate ball [70]. Titration experiments indicate that H-bonding between gelatin and chitosan is responsible for the synergistic effect in the presence of both biopolymers. Adapted with permission from Elsevier, Copyright (2015).

Kovach et al. [70] fabricated a supramolecular calcium phosphate ball by dropping NaH_2_PO_4_·H_2_O precursor solution into CaCl_2_ solution with gelatin and chitosan at 90 °C (pH values ranging from 5.5 to 3.8) under continuous stirring (shown in Figure 5). It has a flower-like structure constructed of thin calcium phosphate platelets generated by the synergistic effect of both the gelatin and chitosan. In the presence of only gelatin, cube-shaped aggregates and large rhombic crystals are formed and can be observed under the light microscope. On the molecular level, the gelatin cannot control the growth of the thin platelets. In presence of only chitosan, the thin calcium phosphate platelets (100 nm) are arranged and form equilateral cubes. Chitosan guided the crystal growth of calcium phosphate and induced bundle formation. Gelatin–chitosan–water complexes influenced the supramolecular ordering and guided calcium phosphate crystal growth to form nano-porous supramolecular structures.

### 2.3. Bioactive Glass

Silicate bioceramics are considered to promote bone regeneration as potential materials for bone repair [32]. This material provides proper mechanical properties, degradation, and manufacturability [71]. Bioactive glass is composed of silica and phosphate. In the organism, it forms a hydroxyapatite layer on the surface, connects with bone cells, promotes the absorption and release of growth factors, and assists in the proliferation and differentiation of osteoblasts [72]. Bioceramic particles composed of silica and titanium oxide composites can promote MG63 cell growth significantly and keep the pH value and ion concentrations of the environment stable. These particles show potential for bone-regenerating applications [73].

**Figure 6:**
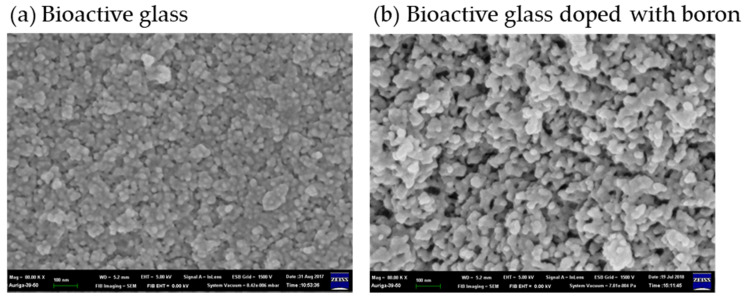
The microstructures of (**a**) bioactive glass and (**b**) bioactive glass doped with boron [74]. Adapted with permission from MDPI AG, Copyright (2020).

Bioactive glass particles usually have mesoporous structures and are advantageous to use as drug delivery systems in dentistry. Bioactive glass can be doped with boron to increase its bioactivity and give it a more porous structure. It is advantageous for drug delivery given its ability for drug adsorption [74] (shown in Figure 6). Mesoporous silica nanospheres were developed as engineered nanocarriers within a foam matrix for long-term and sequential delivery. The matrix was shelled by biopolymers, polylactic acid, or polyethylene glycol via electro-spraying to fuse it with the collagen foam. The biopolymer shell substantially prolonged the release period of bovine serum albumin. It is effective for sequential protein delivery. The foam scaffolding of the nanocarrier system is a potential therapeutic three-dimensional matrix for cell culture and tissue engineering [75].

Bioactive glass was first made by Larry L. Hench in the late 1960s [76] and called bioglass 45S5. The manufacturing method was like that of industrial glass, which is melted at a high temperature of about 1400 °C. However, it was replaced by the sol-gel method to retain the bioactivity of bioglass [77]. The advantages of the sol-gel method are that the synthesis temperature is relatively low, large-scale vacuum processing equipment is not required, the composition ratio can be adjusted, organic and inorganic substances are allowed to be doped, and the uniformity of the product’s particle size can be controlled to obtain high-purity samples with extremely high uniformity [78]. The sol-gel method uses chemically active compounds as precursors. The reactants and precursors can be uniformly dispersed in the liquid environment. Through the control of environmental factors such as temperature, concentration, and pH, the solution can gradually form a sol system, and colloidal particles gradually aggregate and form a polymer structure to create a three-dimensional gel structure.

The sol-gel method can be used to prepare silica-based bioglass for bone tissue regeneration [78]. First, the precursor (i.e., tetraethyl silicate), catalyst solvent, and water are mixed into a homogeneous sol solution. The precursor can be processed under the catalyst, hydrolysis, and polycondensation reactions. Since the precursors of the reaction are dissolved in aqueous solutions of different pH values, the alkoxy groups of the reaction precursors can react with the hydroxyl groups of water. Then, the polycondensation reaction consists of two forms, namely water condensation and alcohol condensation, respectively.

**Figure 7:**
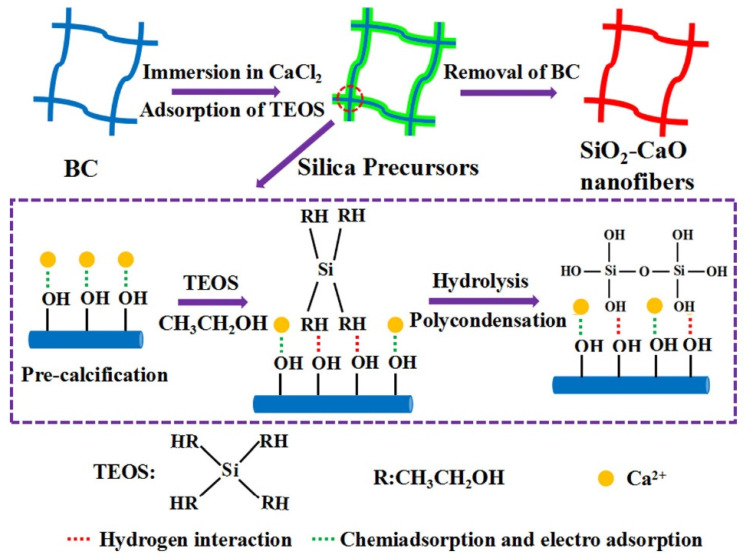
The fabrication process of SiO_2_-CaO binary glass scaffolds [79]. Tetraethyl silicate (TEOS) was polymerized on bacterial cellulose (BC) nanofiber biopolymer and was calcinated to remove the BC template to yield pure silica-based nanofibers. Adapted with permission from Elsevier, Copyright (2017).

Luo et al. [79] fabricated SiO_2_-CaO binary glass scaffolds via bacterial cellulose, nanofibrous biopolymer (shown in Figure 7). Bacterial cellulose was composed of 2.5% (*w*/*v*) glucose, 0.75% (*w*/*v*) yeast extract, 1% (*w*/*v*) tryptone, and 1% (*w*/*v*) Na_2_HPO_4_. It was used as a template for nanofibrous structures. Then, the sol-gel process was used to form fibrous silica-based scaffolds upon bacterial cellulose fibers. The bacterial cellulose template was moved via calcination at 700 °C for 6 h. The fiber diameter of pristine bacterial cellulose ranges from 25 to 95 nm and the scaffold exhibits the dominant mesopores at around 10.6 nm.

## 3. Biopolymers Hybrid Particles Used in Dentistry

In periodontal regeneration surgery, periodontal regeneration membranes are often used to block the invasion of bacteria or fibroblasts. Periodontal regeneration membranes have a porous structure to prevent bacteria or fibroblasts from invading while allowing nutrients and blood to pass through [80,81]. It can create a space to guide the regrowth of periodontal tissue cells. Periodontal regeneration membrane materials are divided into two types: absorbable [82] and non-absorbable [83]. The non-absorbable regeneration membrane must be removed 4–6 weeks after surgery, which is very disruptive. Therefore, artificial synthetic or native materials which are absorbable and have good biocompatibility are often used [9,84]. Various biopolymers can be used, such as hyaluronic acid [85], alginate [86], polylactic acid [87], and chitosan [88].

Hyaluronic acid is a biomolecule composed of glycosaminoglycan (polysaccharide, carbohydrate). In oral tissues, native hyaluronic acid is mainly present in gingiva and the periodontal ligaments. The viscosity, elasticity and hydrophilicity of hyaluronic acid are usually modified to enhance its resistance to degradation [89]. Hyaluronic acid-gelatin hydrogel polymers, β-tricalcium phosphate, and biphasic calcium phosphate ceramics can be fabricated using freeze-drying methods to develop a dental-bone substitute for bone regeneration. This biopolymer and bioceramic mixing scaffold showed excellent bone formation via osteocytes without tissue collapse [85].

Alginate is a natural biopolymer found in seaweed and widely used in the food and pharmaceutical industry. Alginate hydrogel can be developed as a promising scaffold for dental-derived stem cells with a high capacity for osteo-differentiation and adipo-differentiation in vitro [90]. However, pure alginate exhibits low cell adhesion and poor mechanical properties [91]. Bioactive glass containing Zinc and Magnesium was incorporated into alginate networks to improve antibacterial and biological activity, as well as mechanical properties [86]. This shows the potential use-value of the biopolymers of hybrid bioactive glass particles in dentistry.

Periodontal regeneration film is often mixed with other materials to expand its functionality; bioglass can promote tissue regeneration due to its good biocompatibility, and the alkaline environment generated during its degradation can inhibit bacterial growth. However, not all polymers can be combined with bioglass. For example, polylactic acid is often used as a material for periodontal regeneration film because of its good stability, biodegradability, mechanical material, and biocompatibility, and since the product of polylactic acid degradation is acidic, it has a sterilization function. However, after being mixed with bioglass, it will neutralize the alkaline environment produced by the bioglass, causing the periodontal film to form a neutral environment in which bacteria may proliferate [87].

Chitosan has become a novel choice and has the advantages of biodegradability, but the product is neutral after degradation, so the alkaline antibacterial environment of bioglass can continue to function [88]. Moreover, the introduction of bioglass into chitosan increases the hardness and elasticity of the composite film so that the film can still maintain proper mechanical strength in a humid environment. Bioglass can effectively precipitate calcium and phosphorus on the surface of the composite membrane. This type of material has the potential to induce bone regeneration and is significant for promoting the proliferation and metabolism of human periodontal ligament cells. Although there is only a significant increase in the metabolism of human bone marrow stromal cells, it has a significant effect on the mineralization of the two types of cell matrices. Hydroxyapatite nanoparticles (nHA) incorporated in the chitosan/gelatin/nHA scaffolds can promote the deposition of nanocrystalline mineralized tissue inside the cell-seeded scaffolds without the need for exogenous addition of inductive factors [92]. The chitosan hybrid hydroxyapatite membrane also can be designed as a smooth–rough asymmetric structure in the guided regeneration of the barrier membrane for periodontal tissue regeneration [93]. The bilayer collagenous coated chitosan membrane had higher proliferation/metabolic activity compared to the pristine chitosan membranes and the potential for guided bone regeneration to promote bone formation [94]. 

**Figure 8:**
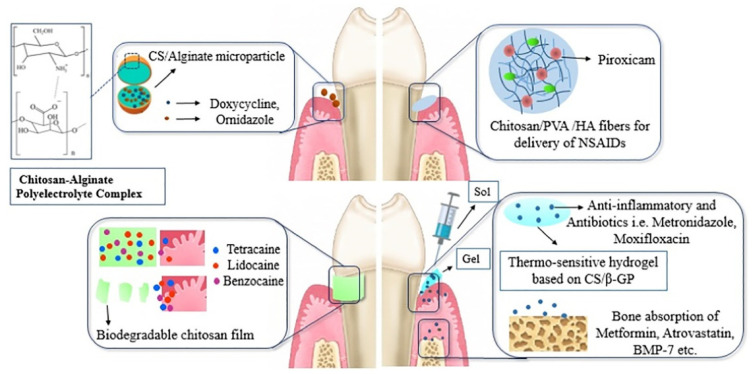
Various drug delivery systems for the treatment of periodontitis [95]. Adapted with permission Elsevier, Copyright (2020).

Moreover, chitosan can be made as micro-/nanoparticles, fibers, film sponges, gels and injectable devices to develop local drug delivery systems for the treatment of periodontitis, tooth caries, or root canal procedures (endodontics). It sustains the release of drugs in the periodontal pockets and therapeutic concentrations for long periods of treatment time [95] (shown in Figure 8). Drug-loaded chitosan micro/nanoparticles can be modified with anionic biopolymers (i.e., alginate) via polyelectrolyte complexation. They have less toxicity and enhance the controlled release in acidic environments. Chitosan can be made as nano/microfibers, but its mechanical property must be improved by composite formulations (i.e., polyvinyl alcohol, PVA). Non-steroidal anti-inflammatory drugs (NSAIDs) can be incorporated in chitosan/PVA fibers for drug delivery systems to reduce inflammation. Biodegradable chitosan films were made via solvent casting for the delivery of effective concentrations of local anesthetics, such as tetracaine, lidocaine, and benzocaine. They can provide prolonged anesthetic treatments and relieve pain. Thermosensitive hydrogels based on chitosan and glycerophosphate were developed as an injectable loaded with anti-inflammatory and antibiotics (i.e., metronidazole, and moxifloxacin) or growth factors (i.e., metformin and bone morphogenetic proteins, BMP-7) to enhance biomineralization and induce bone tissue regeneration.

## 4. Conclusions and Outlook

This review compiles currently available information regarding aspects of biopolymer hybrid particles, which are important in dentistry. Furthermore, information regarding factors affecting biopolymer gel-assisted synthesis for inorganic particles used in dentistry is discussed. From a clinical point of view, calcium phosphate including hydroxyapatite is non-toxic, osteoconductive, and bioactive, both in vitro and in vivo. For a long time, hydroxyapatite was the main material used for bone implants due to its physicochemical properties and bioactivity. However, bioactive glass is more applicable and multifunctional than hydroxyapatite and is currently used in clinical practice and commercially available. In addition, chitosan can be used as a tunable and biodegradable carrier for drug delivery systems. Bioactive glass and chitosan, therefore, present novel options for the design of biopolymer hybrid particles for therapeutic dentistry procedures to promote tissue regeneration. With this review article, we hope to encourage the development of biopolymer hybrid particles use in dentistry, considering their physicochemical properties, bioactivity, and multifunctionality.

## Data Availability

No new data were created or analyzed in this study. Data sharing is not applicable to this article.
